# Molecular Identification of Bacteria Isolated from Marketed *Sparus aurata* and *Penaeus indicus* Sea Products: Antibiotic Resistance Profiling and Evaluation of Biofilm Formation

**DOI:** 10.3390/life13020548

**Published:** 2023-02-16

**Authors:** Mohammad A. Abdulhakeem, Mousa Alreshidi, Fevzi Bardakci, Walid Sabri Hamadou, Vincenzo De Feo, Emira Noumi, Mejdi Snoussi

**Affiliations:** 1 Department of Biology, College of Science, University of Ha’il, Ha’il P.O. Box 2440, Saudi Arabia; 2 Molecular Diagnostics and Personalized Therapeutics Unit, University of Hail, Hail P.O. Box 2440, Saudi Arabia; 3 Department of Pharmacy, University of Salerno, Via Giovanni Paolo II, 132, Fisciano, 84084 Salerno, Italy; 4 Laboratory of Genetics, Biodiversity and Valorization of Bio-Resources (LR11ES41), Higher Institute of Biotechnology of Monastir, University of Monastir, Avenue Tahar Haddad, BP74, Monastir 5000, Tunisia

**Keywords:** *Sparus aurata*, *Penaeus indicus*, 16S RNA sequencing, exoenzymes, antibiotics, biofilm formation

## Abstract

Background: Marketed fish and shellfish are a source of multidrug-resistant and biofilm-forming foodborne pathogenic microorganisms. Methods: Bacteria isolated from *Sparus aurata* and *Penaeus indicus* collected from a local market in Hail region (Saudi Arabia) were isolated on selective and chromogenic media and identified by using 16S RNA sequencing technique. The exoenzyme production and the antibiotic susceptibility patterns of all identified bacteria were also tested. All identified bacteria were tested for their ability to form biofilm by using both qualitative and quantitative assays. Results: Using 16S RNA sequencing method, eight genera were identified dominated by *Vibrio* (42.85%), *Aeromonas* (23.80%), and *Photobacterium* (9.52%). The dominant species were *V. natrigens* (23.8%) and *A. veronii* (23.80%). All the identified strains were able to produce several exoenzymes (amylases, gelatinase, haemolysins, lecithinase, DNase, lipase, and caseinase). All tested bacteria were multidrug-resistant with a high value of the multiple antibiotic index (MARI). The antibiotic resistance index (ARI) was about 0.542 for *Vibrio* spp. and 0.553 for *Aeromonas* spp. On Congo red agar, six morphotypes were obtained, and 33.33% were slime-positive bacteria. Almost all tested microorganisms were able to form a biofilm on glass tube. Using the crystal violet technique, the tested bacteria were able to form a biofilm on glass, plastic, and polystyrene abiotic surfaces with different magnitude. Conclusions: Our findings suggest that marketed *S. aurata* and *P. indicus* harbor various bacteria with human interest that are able to produce several related-virulence factors.

## 1. Introduction

Seafood has great nutritional benefits and economic importance; thus, the bacterial species present in seafood must be identified and studied to determine the best health practices to prevent seafood-borne illnesses [1]. Fish is the food category mainly associated with foodborne outbreaks, accounting for approximately 6–8% of the total food-borne diseases. This prevalence is greater than the incidence of food illness cases from chicken and beef [2]. Both pathogenic and harmful bacteria can be introduced into seafood products during the manufacturing process and in the supply chain [3,4]. Shellfish are considered as a major source of seafood-borne pathogens in humans, as they are usually consumed undercooked or raw. Water warming due to climate change has recently become an issue as it would elevate the microbial population, including *Vibrio* species in particular foodborne strains and other pathogenic bacteria that ultimately end up in seafood environments, inducing more seafood-borne diseases as a result of the intake of contaminated seafood. Fish and other seafood are a source of various microorganisms with human health interest including Gram-negative bacteria (*Pseudomonas*, *Shewanella*, *Psychrobacter*, *Pseudoalteromonas*, *Moraxella*, *Acinetobacter*, *Flavobacterium*, *Vibrio*, *Photobacterium*, and *Aeromonas*) and Gram-positive bacteria (lactic acid bacteria (LAB), *Micrococcus*, *Corynebacterium*, *Vagococcus*, *Bacillus*, and *Clostridium*) [5]. Pathogenic bacteria in seafood can be transmitted to humans during food intake, inducing serious health issues, including cellulitis and septicemia. Pathogens can also enter the bloodstream through wounds or open cuts while handling infected seafood or swimming, causing necrotizing fasciitis and fatal septicemia in susceptible individuals [6,7]. The high incidence of seafood poisoning indicates substantial challenges in controlling pathogenic microbes that induce food-borne illnesses [8,9]. The microbial diversity in fish is highly related to the conditions in which they live and remain after harvesting. Microbial diversity can be determined by a wide range of parameters, including location, origin, water type (e.g., brackish or freshwater), and catching and handling processes [10]. Understanding the prevalence, ecology, concentration, and dynamics of pathogenic and spoilage microorganisms in seafood would contribute to developing effective preservative mechanisms. Antibiotics are widely used in aquaculture for prophylactic and therapeutic purposes. However, the misapplication of antibiotics has significantly contributed to the increase in antibiotic-resistant microbes (ARMs) and resistance genes in aquaculture farms and neighboring coastal settings [11]. In addition, the excessive use of antimicrobials could lead to widespread multidrug-resistant microorganisms in fish, shellfish, and their surrounding water [12,13,14,15,16].

The prevalence of ARMs has become a worldwide issue in all food plants, not only in seafood. ARMs have become a major concern for public health, and many isolates from seafood have demonstrated a higher degree of resistance against a wide range of antibiotics [17]. 

Hence, the main objective of this study was to identify the main bacteria in *Sparus aurata* and *Penaeus indicus*, which are highly consumed in the Ha’il region. Further importance was given to the determinants of antibiotic resistance and biofilm formation in the identified isolates. The current study also investigated the ability of these isolates to produce different enzymes. It was hypothesized that the isolated bacteria would have different degrees of antibiotic resistance and biofilm formation, as well as different exoenzyme profiles.

## 2. Materials and Methods

### 2.1. Sampling Material and Bacterial Isolation

Bacteria were isolated from gilthead sea bream (*Sparus aurata* L.) and shellfish (*Penaeus indicus* H. Milne-Edwards). These samples were obtained from a local market in Hail region-Saudi on 25 February 2022. Fish with red spots on their skin were targeted, as this is an indication of microbial infection. Upon arrival, gilthead sea bream and prawns were immediately washed using sterile seawater, gutted, headed, and shucked with a sterile knife. Twenty-five grams from prawn abdomen meat, and the intestines, gills, and muscle meat from *S. aurata* were enriched in 225 mL of alkaline peptone water supplemented with 1% NaCl [18]. The inoculated broth media was incubated overnight at 37 °C. After incubation, a loopful from each enrichment culture was steaked onto thiosulfate–citrate–bile salt–sucrose agar (TCBS) (Agar; Sigma Aldrich, Darmstadt, Germany) and onto *Vibrio* ChromoSelect agar (Sigma Aldrich, Germany), before incubating for 18 to 24 h at 37 °C.

### 2.2. Bacterial Identification and Phylogenetic Analyzes

Twenty-one bacterial isolates were selected from both selective and chromogenic media (seven isolates from *P. indicus* abdomen muscles, six isolates from *S. aurata* intestines, four isolates from *S. aurata* muscle meat, and four isolates from *S. aurata* gills). The DNA extraction was conducted using the Wizard Genomic DNA Purification Kit (Promega, Madison, WI, USA). The polymerase chain reaction (PCR) was performed using Applied Biosystems^TM^ (Waltham, MA, USA) apparatus, with the following PCR conditions: initial denaturing step for 5 min, 30 cycles of amplification (30 s denaturation at 95 °C, 30 s annealing step at 52 °C, and 30 s extension at 72 °C), and a final extension step at 72 °C for 5 min. After PCR, the obtained products of the 27F-907R regions were electrophoresed using 2% agarose gel (110 V, 150 mA, 45 min). It was observed that the size of the products was between 1200–1400 bp. Then, the cleanup phase was started using ExoSAP-IT Express PCR Cleanup Reagents. For the experiment, 10 µL of each PCR product was mixed with 4 µL of the cleanup reagent.

Molecular identification of isolated bacteria was achieved by sequencing the 16S rDNA gene using the universal primer 27F (5′–AGAGTTTGATCMTGGCTCAG–3′) and the reverse primer 907R (5′–CCGTCAATTCCTTTRAGTTT–3′) previously described by Muyzer et al. [19]. After the DNA sequencing reaction was completed, the sequencing products were purified using the gel filtration method with Sephadex. After the purification process, the DNA sequencing process was started. This was performed on the ABI 3130XL device using the capillary electrophoresis method.

Sequence alignment was performed using BioEdit version 7.1.3.0 [20], and a total of 859 bp were successfully aligned in the final dataset consisting of 31 nucleotide sequences, including *Salmonella enterica* as the outgroup. The 16S rDNA sequences were subjected to a BLAST search to determine the sequence homology with the sequences previously deposited in the NCBI to identify isolated bacterial species and strains. The sequences with the highest homology belonging to *Vagococcus fluvialis*, *Staphylococcus aureus*, *Staphylococcus epidermidis*, *Shewanella indica*, *Photobacterium damselae*, *Morganella morganii*, *Bacillus cereus*, *Aeromonas veronii*, and *Vibrio harveyi* were added to our dataset to determine the relation of our isolates with them. A phylogenetic tree was generated by the neighbor-joining method [21] with a bootstrap test (1000 replicates) [22]. The Kimura two-parameter method [23], the best-fitting model for our sequence dataset, was used to compute the pairwise evolutionary distances among the sequences, with the gaps removed by the pairwise deletion option implemented in MEGA 11 software [24].

### 2.3. Exoenzyme Production 

The identified isolates were tested for their abilities to produce several exoenzymes including DNAase, lipase, amylase, caseinase, and lecithinase, according to the protocol described by Hörmansdorfer et al. [25] and Snoussi et al. [26]. For the experiment, PBS agar medium was supplemented with Tween-80 (lipase activity), starch (amylase activity), skim milk powder (caseinase activity), and egg yolk (lecithinase production). Petri dishes were incubated for 72 h at 37 °C, and the formation of a clear zone around the inoculated spots was considered a positive test. In addition, hemolysin production was tested on human blood agar supplemented with 5% human blood (Oxoid Ltd., Basingstoke, UK) [26]. 

### 2.4. Antibiotic Susceptibility Test 

Susceptibility to several antimicrobial agents was determined using the disc diffusion assay on Mueller–Hinton agar/1% NaCl [14,27]. The following antibiotics (Oxoid, UK) were tested against all identified bacteria: amikacin (AK, 30 μg), ampicillin (AMP, 10 μg), gentamicin (GEN, 10 μg), tetracycline (TET, 10 μg), ertapenem (ETP, 10 μg), fosfomycin (FOS, 200 μg), norfloxacine (NOR, 10 μg), linezolid (LZD, 30 μg), nitrofurantoin (F, 100 μg), ciprofloxacin (CIP, 5 μg), nalidixic acid (NA, 30 μg), moxifloxacin (MXF, 30 μg), meropenem (MEM, 10 μg), ticarcillin (TIC, 75 μg), piperacillin + tazobactam (PPT, 75/10 μg), cefotaxime (CTX, 30 μg), tigecycline (TGC, 15 μg), pristinamycin (PTN, 15 μg), rifampicin (RAM, 30 μg), erythromycin (E, 15 μg), chloramphenicol (C, 30 μg), amoxicillin + clavulanic acid (AUG, 30 μg), temocillin (TMO, 30 μg), tobramycin (TOB, 10 μg), sulphamethoxazle + trimethoprim (SXT, 25 μg), ceftazidime (CZD, 30 μg), ceftaroline (CPN, 5 μg), colistin (CST 50 μg), netilmicin (NET, 30 μg), and teicoplanin (TEC, 30 μg). After incubation at 37 °C for 18 to 24 h, the diameter of the inhibition zone was measured using a 1 mm flat rule. The antibiotic susceptibility profile of the isolate was interpreted as sensitive, intermediate, and resistant according to the Clinical and Laboratory Standards Institute (CLSI) M45 and (CLSI) M100 guidelines Institute [28,29]. Two mathematic indices were used to interpret the results obtained: (i) the antibiotic resistance index (ARI) of each bacterial population [30], and (ii) the multiple antibiotic resistances (MAR) index of the isolates [31].

### 2.5. Adhesion Properties and Biofilm Formation Screening

#### 2.5.1. Exopolysaccharide (Slime) Production

The ability of all identified bacteria to secrete an exopolysaccharide layer (slime production) was tested using the same protocol previously described by Snoussi et al. [32] adapted to *Vibrio* species. Colonies obtained on Congo red agar were interpreted as slime producers (pigmented colonies), while unpigmented colonies were interpreted as slime nonproducers [33].

#### 2.5.2. Wolfe Test

The ability of the identified bacteria to adhere to the glass surface was tested using the same protocol described by Wolfe et al. [34]. For the experiment, a 10 mL glass tube (0.5 cm in diameter) containing seawater broth (5 g of Bactotryptone, 3 g of yeast extract, and 3 mL of glycerol in 700 mL of seawater and 300 mL of purified water) was used to grow overnight all bacteria at 37 °C. Afterward, 100 μL of this pre-enriched culture were added to inoculate new tubes containing the same medium and incubated at 37 °C for 10 h without shaking. Following a 15 min staining period with 1% (*w*/*v*) crystal violet, all glass tubes were cleaned with distilled water before being used for further testing. Glass-biofilm positives were bacteria that produced a purple pellicule on the cultures’ air surface.

#### 2.5.3. Biofilm Formation on Polystyrene Microtiter Plates

The capacity of bacteria to create a biofilm on 96-well polystyrene microtiter plates was estimated using the Toledo-Arana et al. protocol [35]. Brain Infusion Broth (BHI/0.25% (*w*/*v*) glucose) medium was used for the pre-enrichment of all bacterial strains. After overnight culture of the tested bacterial in a microtiter plate for 24 h at 37 °C, adherent bacteria were stained using crystal violet (1%) for 15 min, and then solubilized with ethanol–acetone (80:20 *v*/*v*). The optical density (OD_595nm_) was measured spectrophotometrically. Bacteria were interpreted as (−) non-biofilm forming OD_595_ ≤ 1, (+) weak biofilm forming 1 < OD_595_ ≤ 2, (++) medium biofilm forming 2 < OD_595_ ≤ 3, or (+++) strong biofilm forming OD_595_ > 3 [33]. Each essay was performed three times. 

#### 2.5.4. Biofilm Formation on Glass and Plastic Surfaces

Glass material (circular 12 mm diameter cover glasses) and a plastic surface (12 mm diameter) were used for the quantitative estimation of biofilm-forming capacities of all identified strains from *S. aurata* and *P. indicus* inserted into the bottom of 24-well (15 mm diameter each well) microtiter plates and filled with 2 mL of each bacterial suspension (10^9^ UFC/mL in PBS) for 24 h at 37 °C. The experiments were carried out in triplicate and three times. The same procedure described by Henriques et al. [36] was followed using 600 μL of crystal violet for 5 min to stain the biofilm-forming bacteria fixed on the abiotic surfaces selected. The pieces were gently washed in water and dried before being immersed in 1 mL of 33% (*w*/*v*) acetic acid to release and dissolve the stain. Using a microtiter plate reader, the OD of the resulting solution was measured at 570 nm (Bio-Tek, Model Synergy HT, city). Results were interpreted using the scheme proposed by Stepanović et al. [37], where bacteria were interpreted as nonadherent (0) OD ≤ ODc, weakly adherent (+) ODc < OD ≤ 2 × ODc, moderately adherent (++) 2 × ODc < OD ≤ 4 × ODc, or strongly adherent (+++): 4 × ODc < OD. 

### 2.6. Statistical Analysis

All experiments were performed in triplicate, and the average and standard deviation were calculated using the SPSS 25.0 statistical package for Windows. 

## 3. Results

### 3.1. Morphological Characterization and 16SRNA Identification of Bacterial Isolates 

The analysis of different samples from *P. indicus* on TCBS agar medium revealed the characterization of two morphotypes: yellow colonies of 1 to 2 mm in diameter and green-yellow colonies with a diameter of about 2–3 mm, respectively. In addition, on *Vibrio* ChromoSelect agar, four different morphotypes based on color and size were observed (Table 1). Similarly, 14 morphotypes were obtained from *S. aurata* after being cultured on TCBS and *Vibrio* ChromoSelect agar medium. The dominant color on TCBS agar plates was yellow (diameter between 1 and 7 mm). However, on *Vibrio* ChromoSelect agar, nine isolates with various ranges of colony shapes, sizes, and colors were seen, including blue, turquoise, purple, pink, light green with a green center, and colorless colonies (Table 1). 

**Figure 1:**
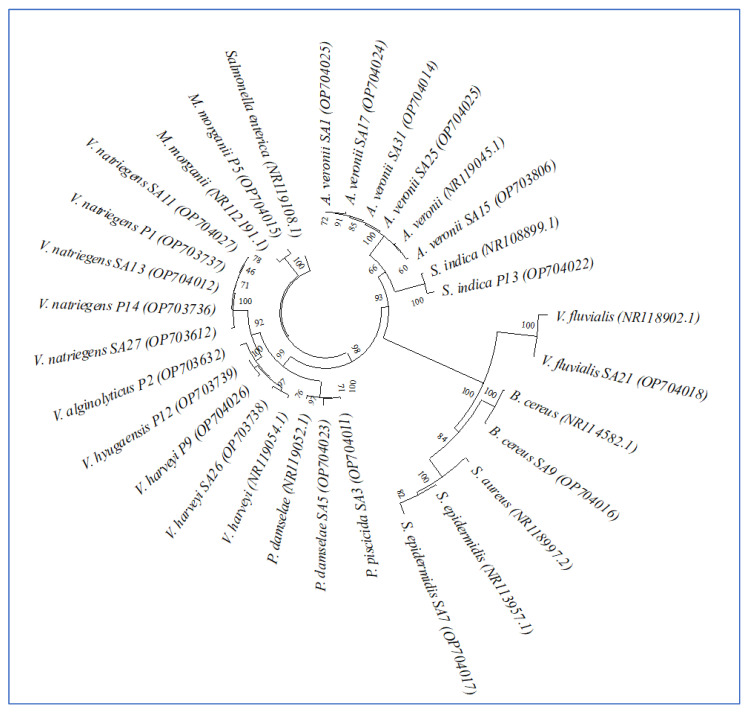
Phylogenetic tree of the 21 identified bacteria from *P. indica* and *S. aurata* based on their 16S RNA gene sequences obtained using the UPGMA method.

Twenty-one colonies were subsequently analyzed using molecular techniques (16S RNA) and bioinformatics (BioEdit software and National Center for Biotechnology Information (NCBI)) to certain the identity of these isolates. The results demonstrated that the main bacteria identified in both samples belonged to the *Vibrio* genus with four different species, including *V. natriegens*, *V. harveyi*, *V. alginolyticus*, and *V. hyugaensis*. The second most dominant species was *Aeromonas veronii*. The phylogenetic tree (Figure 1) shows the genetic relationship of the 21 identified bacterial strains, isolated from various organs of *S. aurata* and shrimps (*P. indicus*) using the NJ method. 

### 3.2. Exoenzyme Production 

The bacteria identified in this study were investigated for their capability to produce several hydrolytic enzymes. The results demonstrated that all tested bacteria were able to produce amylase (100%), but other enzymes were only induced by some bacteria. The percentage of the total bacteria secreted by each enzyme was as follows: lipase (80.95%), DNase (71.42%), caseinase (66.66%), lecithinase (57.14), hemolysins (52.38), and gelatinase (47.61%) (Table 2). Some bacteria were able to produce all enzymes tested, such as *V. harveyi* (P9), *V. alginolyticus* (P2), *A. veronii* (SA15), *V. fluvialis* (SA21). It was observed that some bacteria with the same identify had different exoenzyme profiles. 

### 3.3. Antibiotic Susceptibility Test

**Figure 2:**
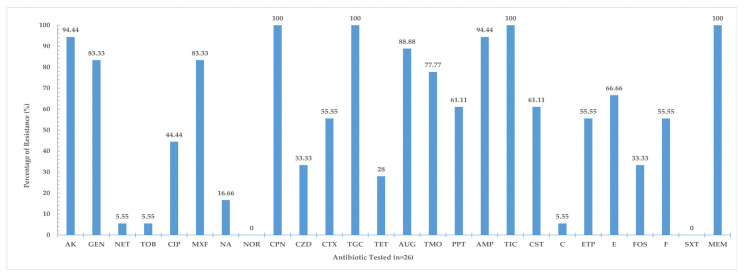
Percentage resistance (%) of 18 Gram-negative bacteria isolated from *S. aurata* and *P. indicus* to 26 antibiotics.

The results of the antibiotic susceptibility test showed that some of the tested bacteria were resistant to all antibiotics used with the exception of norfloxacin and trimethoprim–sulfamethoxazole which were effective against all Gram-negative bacteria (Supplementary Material Table S1). The analysis indicated that all Gram-negative bacteria were completely resistant to tigecycline, ceftaroline, meropenem, and ticarcillin and highly resistant to amikacin (94.44%), ampicillin (4.44%), amoxicillin + clavulanic acid (88.88%), gentamicin (83.33%), and moxifloxacin (83.33%) (Figure 2). The lowest percentage of resistance was recorded for the following antibiotics: norfloxacin (0%), trimethoprim–sulfamethoxazole (0%), tobramycin (5.55%), netilmicin (5.55%), chloramphenicol (5.55%), and nalidixic acid (16.66%). 

**Figure 3:**
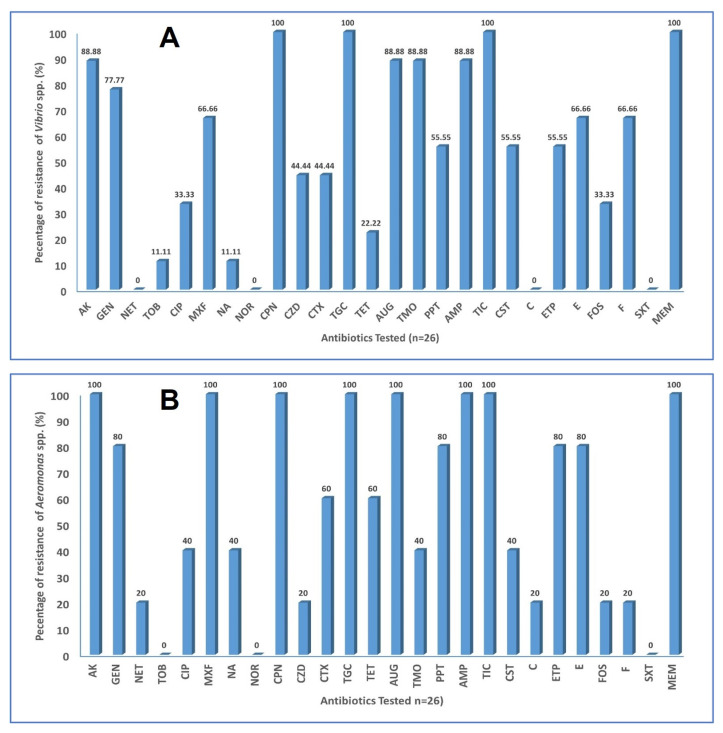
Percentage resistance of *Vibrio* spp. (**A**) and *Aeromonas* spp. (**B**) strains to the 26 antibiotics tested in this study.

In addition, it is worth noting that *Vibrio* spp. (n = 9) were particularly completely resistant to ceftaroline, tigecycline, ticarcillin, colistin, and meropenem (Figure 3). On the other hand, these bacteria were found to be sensitive or dose-dependently resistant (intermediate) to netilmicin, norfloxacin, chloramphenicol, and trimethoprim–sulfamethoxazole. Similarly, the *Aeromonas* spp. (n = 5) strains were completely resistant to amikacin, moxifloxacin, ceftaroline, tigecycline, amoxicillin–clavulanic acid, ampicillin, ticarcillin, and meropenem. The lowest percentage of resistance was recorded with three antibiotics (tobramycin, norfloxacin, and trimethoprim–sulfamethoxazole) (Figure 3).

Overall, the tested bacteria could be considered as multidrug-resistant microorganisms, as all isolates were resistant to three or more antibiotics from different classes (Supplementary Tables S1 and S2). In fact, the multiple antibiotic resistance index (MARI) for *Vibrio* spp. (n = 9) ranged from 0.384 (*V. harveyi* P9) to 0.653 (*V. alginolyticus* P2). Regarding the *Aeromonas* spp. strains (n = 5), the MARI ranged from 0.461 (*A. veronii* SA17) to 0.692 (*A. veronii* SA25). The MARI ranged from 0.423 (*M. morganii* P5) to 0.653 (*S. indica* P13) for the other Gram-negative identified bacteria. Similarly, for Gram-positive bacteria, the MARI was about 0.5 for *B. cereus* SA9, 0.333 for *S. epidermidis* SA7, and 0.277 for *V. fluvialis* SA21. In addition, our results revealed that the calculated ARI varied from 0.542 for all *Vibrio* strains (n = 9) to 0.553 for all *Aeromonas* strains (n = 5). Taken together, the ARI for the 18 Gram-negative bacteria was about 0.544, while the same index was lower (ARI = 0.462) for the Gram-positive bacteria tested (Table 3).

### 3.4. Slime Production on CRA Plates and Glass Tubes (Wolfe Test)

**Figure 4:**
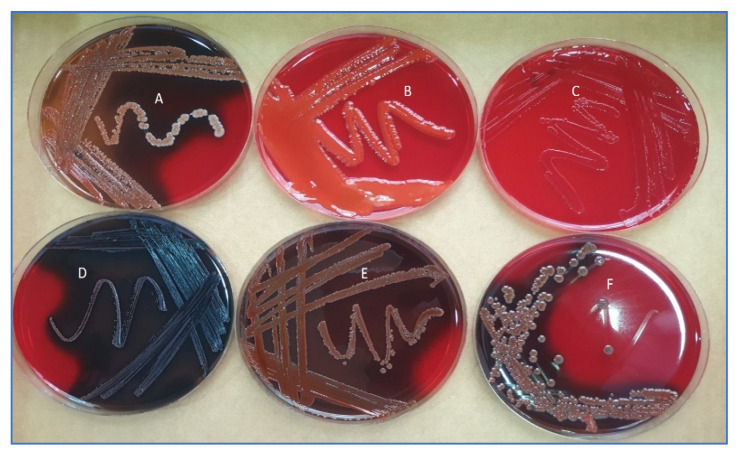
Different morphotypes described on Congo red agar plates: (**A**) white with red center; (**B**) orange; (**C**) red; (**D**) black; (**E**) Bordeaux; (**F**) black-gray.

The phenotypic production of slime was assessed by culturing the bacteria on Congo Red Agar plates. Pigmented colonies were considered as normal slime-producing bacteria, whereas colorless colonies were classified as non-slime-producing. Among the tested isolates, six out of 21 (28.57%) were able to induce slime, indicated by black colonies, and the remaining 15 bacteria were non-slime-producing characterized by red, orange, Bordeaux, white with a red center, or black-gray morphotypes (Figure 4).

**Figure 5:**
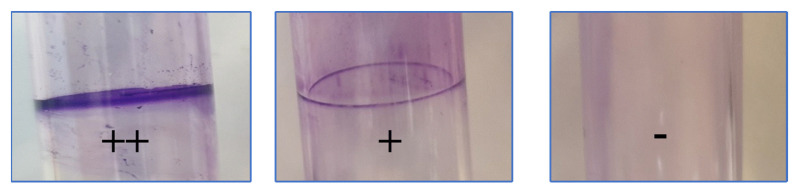
Pellicule formation on the surface of the tested glass tube stained with 1% crystal violet. (++): large pellicule formation; (+) weak pellicule formation; (−) no pellicule formation.

All tasted bacteria were able to adhere to the glass, giving a purple pellicule on the air surface of the glass tube, except for *Photobacterium damselae*. The intensity of the color of the crust formed ranged from intense to moderate. Moreover, 10 bacteria out of 21 tested were strongly adhesive to glass surface (Figure 5). All these data are summarized in Table 4.

### 3.5. Quantitative Estimation of Biofilm Formation by Tasted Bacteria on Abiotic Surfaces

On a polystyrene 96-well microtiter plate (U-bottom), five bacteria (*V. natrigens* SA11, *V. alginolyticus* P2, *V. hyugaensis* P12, *P. piscicida* SA3, and *B. cereus* SA9) out of 21 were medium biofilm forming with an optical density of about 2 < OD_595_ ≤ 3. In addition, 12 bacteria weakly adhered to polystyrene and formed a weak biofilm (1 < OD595≤ 2) on the polystyrene surface (96-well plate). Interestingly, all tested bacteria were able to adhere to glass and plastic surfaces to different degrees, with the exception of only two strains (*V. natrigens* SA11 and *V. hyugaensis* P12) on glass and three strains on plastic, namely, *V. hyugaensis* P12, *A. veronii* SA15, and *A. veronii* SA25, regardless of their origin (*S. aurata* or *P. indicus* samples). In fact, two bacteria isolated from *P. indicus* (*V. natrigens* P14 and *S. indica* P13) and one from *S. aurata* (*V. natrigens* SA11) adhered strongly to the plastic surface, whereas no bacteria were strongly biofilm forming on glass material. Overall, 9/21 (42.85%) formed a biofilm on polystyrene, in contrast to 19/21 (90.47%) on glass and 18/21 (85.71%) on the plastic surface, to different degrees ranging from weak to strong. Interestingly, six bacteria (*V. harveyi* P9, *V. alginolyticus* P12, *A. veronii* SA1, *A. veronii* SA31, *P. piscicida* SA3, and *B. cereus* SA9) were able to adhere to all tested surfaces to different degrees. Lastly, in particular, *B. cereus* (SA9) isolated from the intestines of *S. aurata* was moderately able to adhere to all tested surfaces (polystyrene, glass, and plastic) (Table 5).

## 4. Discussion

The current study identified bacteria in fish and shrimp (*S. aurata* and *P. indicus*) and subsequently investigated their ability to produce different arrays of extracellular enzymes, their biofilm formation ability, and their antibiotic susceptibility. The results identified eight genera of bacteria in the analyzed samples. The abundant bacterial species found were from the *Vibrio* and *Aeromonas* genera, accounting for nine and five species, respectively. These findings consistent with previous publications that showed that *Vibrio*, *Aeromonas* and *Photobacterium* genera were frequently identified in marketed sea food products from around the world [38,39,40,41], as well as in Saudi Arabia [42,43,44,45,46,47,48,49,50]. In fact, Al-Sunaiher and colleagues [42] reported the isolation 62 *Vibrio* spp. strains belonging mainly to *V. hollisae* (54.5%), *V. fluvialis* (20.5%), *V. damselae* (12.6%), *V. alginolyticus* (6.8%), and *V. vulnificus* (4.5%) from *Oreochromis niloticus* L., *O. spilurus* L., *Mugil cephalus* L., *Dicentrarchus labrax* L., *Siganus rivulatus* L., and *Carus gariepinus* L. In 2016, Elhadi and colleagues [44] reported the prevalence of *E. coli* (18.6%), *Enterococci* (14.4%), *Pseudomonas* (14%), and *Salmonella* (16.8%) in imported frozen fish (*Pangasius pangasius*, *Cirrhinus mrigala*, *Oreochromis niloticus*, *Cyprinus carpio*, *Labeo rohita*, *Chanos chanos*, and *Rastrelliger brachysoma*) from a local market in Eastern province of Saudi Arabia. Similarly, using the 16S rDNA technique, Alikunhi and colleagues [43] identified different bacteria from 13 edible fish species from Jeddah province, namely, *P. stutzeri*, *V. harveyi*, *Aeromonas* sp., *A. salmonicida*, *Rahnella aquatilis*, *V. damselae*, *Hafnia* sp., *Pseudoalteromonas* sp., and *Psychrobacter faecalis*.

More recently, Beyari and colleagues [49] studied the bacterial diversity in some marketed fish retails from Jeddah province and reported the identification of 17 different bacterial genera (dominated by *Aeromonas*, *Pseudomonas*, *Psychrobacter*, and *Alcaligens*). The same authors reported the identification of 32 different species including some human pathogenic ones such as *R. aquatilis*, *Proteus vulgaris*, *Klebsiella quasipneumoniae*, *Yersinia enterocolitica*, *P. lundensis*, *P. oryzihabitans*, *Psychrobacter phenylpyruvicus*, *P. sanguinis*, *Alcaligenes faecalis*, and *P. putida*.

The presence of pathogenic bacteria in fish and other seafood is thought mainly to result from the growth conditions, harvesting, and preservation processes that support the spread of microorganisms, particularly pathogens. Therefore, it has been found that cautious processing methods could significantly reduce microbes in fish and other seafood [51]. The major isolated bacteria found in the study were Gram-negative bacteria. This result is similar to previous publications [52]. The main bacteria identified in the current study were *Vibrio* species, accounting for 43% of the total bacteria identified. *Vibrio* species, including *V. harveyi*, *V. alginolyticus*, *V. natriegens*, and *V. hyugaensis* are associated with many human and fish diseases. The next most abundant genus was *Aeromonas veronii* in both fish and shrimp; this bacterium, along with other *Aeromonas* species, has been linked to diarrhea cases in children [53], where approximately 8% of acute enteric infections are induced by *Aeromonas* species [54]. It was found that most *Aeromonas* species could be isolated from different environments, including rivers, meat, and fish, as well as from patients suffering from diarrhea [55,56,57].

Thus, *Aeromonas* species are considered to be primary pathogens in aquaculture that can grow at refrigerator temperature and, hence, can be a major source of food contamination, especially where is a probability of cross-contamination with prepared-to-consume food products [58]. Recently, many fish infections have been initiated by *Aeromonas* species [59,60,61,62]. Other bacterial genera detected in this study include *Shewanella*, *Photobacterium*, *Vagococcus*, *Staphylococcus*, and *Bacillus*. Bacteria can live comfortably in aquatic settings; thus, bacterial infection has become the main barrier to the success of aquaculture farms [63].

This investigation also looked at the ability of the identified bacteria to produce extracellular enzymes, which have been recognized as an indicator of health risk in microbes isolated from different sources, including clinical, food, and environmental samples [64,65]. It was found that the bacterial isolates could yield at least two exoenzymes, including amylase, the only enzyme was produced by all isolates. These results indicated that all isolated bacteria produced a variety of extracellular enzymes, but each isolate had a distinct pattern of hydrolytic enzymes. Enzymes produced by bacteria could potentially modulate the bacterial virulence and pathogenicity, breaking down proteins and making them available for proliferation [66,67]. The secretion of some enzymes and toxins has been found to be responsible for food spoilage and can make the bacteria more resistant to antibiotic agents, leading to therapeutic issues [68]. Amylase was the only enzyme produced by all isolated bacteria, which may indicate the capability of all isolates to use this enzyme to hydrolyze starch [69]. Almost 50% of *Vibrio* isolates in the current study expressed all exoenzymes tested, with complete production of lipase and amylase. In a similar manner, several extracellular enzymes were produced by *Vibrio* [33,68]. Approximately half of the bacterial isolates were capable of producing hemolysin and gelatinase; these enzymes are recognized as virulence factors as both are associated with bacterial pathogenicity [70]. In addition, 80% of the isolates had lipolytic activity, which is associated with the acquisition of nutrients by degrading host lipids. More than 66% of the isolates had DNase and caseinase activities. DNase has a function as an endonuclease and partially plays a role in DNA hydrolysis, whereas caseinase is associated with bacterial pathogenicity. Several exoenzymes have been detected in *Vibrio* bacteria from different sources, including fish, shrimp, and shark [71,72].

The analysis of antibiotic resistance indicated that all Gram-negative bacteria were completely resistant to tigecycline, ceftaroline, meropenem, and ticarcillin. Moreover, *Vibrio* spp. were particularly completely resistant to ceftaroline, tigecycline, ticarcillin, colistin, and meropenem, whereas *Aeromonas* spp. strains were completely resistant to amikacin, moxifloxacin, ceftaroline, tigecycline, amoxicillin–clavulanic acid, ampicillin, ticarcillin, and meropenem. The antibiotic resistant indices (ARIs) for *Vibrio* and *Aeromonas* species were approximately 0.542 and 0.553, respectively. Examination of antibiotic resistance in bacteria isolated from fish and shellfish is essential in order to estimate the level of changes in water ecosystems as a result of human activities [73]. The analysis revealed that the ARI of all the Gram-negative bacteria was 0.544, whereas that of Gram-positive bacteria was 0.305. It has been proposed that bacteria with ARI indices greater than two could be a potential source of high risk where chemicals, including antibiotics, are in high use [74]. These two indices (MARI and ARI) were previously used to study the distribution of multidrug-resistant microorganisms associated with fish and shellfish products [38]. In fact, Al-Sunaiher and colleagues [42] found that *V. vulnificus*, *V. damselae*, *V. fluvialis*, *V. hollisae*, and *V. alginolyticus* species were resistant to several antibiotics tested with high percentages: amoxycillin (66.7–100%), oxytetracycline (33.3–100%), ampicillin (33.3–100%), penicillin (79–100%), chloramphenicol (0–37.5%), sulfonamide (70–100%), cloistin (0–66.7%), tetracycline (6.7–100%), lincomycin (62.5–100%), trimethoprim (20–100%), nalidixic acid (0–63.3%), nitrofurantoin (0–100%), and oxolinic acid (0–100%). Interestingly, the same team reported that the isolated V. vulnificus strains were resistant (100%) to trimethoprim, tetracycline, sulfamethoxazole, penicillin, oxytetracycline, ampicillin, amoxicillin, and lincomycin. Al-Ghanayem and colleagues [50] reported the identification of *Aeromonas* spp., *E. coli*, *Enterobacter* spp., *Proteus* spp., *Enterococcus* spp., and *Streptococcus* spp. strains from the local fish market at Shaqra (Riyadh province, Saudi Arabia) highly resistant to amoxicillin, bacitracin, chloramphenicol, ciprofloxacin, erythromycin, gentamycin, and tetracycline with a multidrug resistance index ranging from 0.33 for *Streptococcus* species to 0.44 for *E.coli* strains.

The result of phenotypic slime production revealed that 28% of the total isolates were positive slime producers. These isolates belong to the *Vibrio* and *Anemones* genera. Slime production is measured as an important virulence factor in some pathogenic bacteria, including *Vibrio* and *Aeromonas* species, and it could be an indicator of a high-risk commination [75]. Slime is used by bacteria as a protective mechanism against external environments; thus, microbes coated with slime are more resistant to antibiotics and other stressors. Slime molecules are considered to be involved in biofilm formation; indeed, they play a significant role in the initial stages of biofilm development [76,77]. Previous studies have reported the characterization of several morphotypes formed by *V. alginolyticus* isolated from fish (*S. aurata*, *D. labrax*) on Congo red agar, and most of them were slime producers with black colonies [32,33]. Similarly, Snoussi and colleagues [16] reported the identification of *A. hydrophila*, *Staphylococcus* spp., *V. alginolyticus*, *Enterobactercloacae*, *K. ornithinolytica*, *K. oxytoca*, and *Serratia odorifera* from seabass, seabream, roseshrimp, and blue mussel. These strains produced five morphotypes based on the colorimetric scale on the tested medium (Bordeaux, red with dark center, pink with red center, pink, and red colonies).

The capacity of the isolates to form biofilms on different materials, including polystyrene, glass, and plastic, was investigated. The analysis indicated that 42.8% of the isolates formed biofilms on polystyrene, in contrast to 90.4% on glass and 85.7 on plastic, to varying degrees ranging from weak to strong. Biofilm development seemed to be affected by surface properties; the use of polystyrene materials is highly recommended to avoid biofilm formation [78]. Some isolates, including *V. alginolyticus*, *A. veronii*, *P. piscicida,* and *B. cereus*, tended to form biofilms on all tested surfaces. The results of this study are similar to others demonstrating that *Aeromonas* and *Vibrio* species from fish and shellfish and their surrounding water were able to form biofilms on different biotic and abiotic surfaces to different degrees [32,33,79]. Bacteria that develop biofilms are greatly resistant to changing environments, including antibiotics and detergents [80,81]. Thus, microbial biofilm development is a topic of important interest in many fields, including food and medical industries, as it is a significant contributor to bacterial virulent, which can lead to serious infections that are difficult to treat [82,83]. The precipitation of mineral and food residues in food manufacturing could positively affect the development of biofilms [84].

## 5. Conclusions

This investigation provided clear evidence that both fish and shrimp collected from local markets, having been initially harvested from an aquaculture farm, had a diversity of bacterial genera. The outcomes of this study revealed that the main bacterial genera identified were *Vibrio* and *Aeromonas*. Antimicrobial resistance was also demonstrated in all bacterial isolates, and high multidrug resistance indices were obtained in most of the tested isolates. The majority of isolates were biofilm producers, suggesting a significant threat from these isolates in the food industry. Therefore, control and prevention of microbial contamination must be taken into consideration in order to obtain healthy and uncontaminated food, particularly seafood.

## Figures and Tables

**Table 1:** The 16S RNA identification results and morphological characteristics of the 21 bacterial isolates from *P. indicus* and *S. aurata* obtained from TCBS and Vibrio ChromoSelect agar medium.

Agar	Site of Isolation	Colony Color	Code	Bacteria Name	Accession
TCBS agar	*P. indicus* abdomen muscles	Yellow	P_12_	*Vibrio hyugaensis*	OP703739.1
	*P. indicus* abdomen muscles	Green yellow	P_13_	*Shewanella indica*	OP704022.1
	*P. indicus* abdomen muscles	Yellow	P_14_	*Vibrio natriegens*	OP703736.1
*Vibrio* ChromoSelect agar	*P. indicus* abdomen muscles	Blue	P_1_	*Vibrio natriegens*	OP703737.1
	*P. indicus* abdomen muscles	Turquoise	P_2_	*Vibrio alginolyticus*	OP703632.1
	*P. indicus* abdomen muscles	Colorless	P_5_	*Morganella morganii*	OP704015.1
	*P. indicus* abdomen muscles	Purple	P_9_	*Vibrio harveyi*	OP704026.1
TCBS agar	*S. aurata* intestines	Yellow	SA_1_	*Aeromonas veronii*	OP704025.1
	*S. aurata* intestines	Yellow	SA_3_	*Photobacterium. Piscicida*	OP704011.1
	*S. aurata* muscles	Yellow	SA_17_	*Aeromonas veronii*	OP704024.1
	*S. aurata* muscles	Blue green	SA_21_	*Vagococcus fluvialis*	OP704018.1
	*S. aurata* gills	Yellow	SA_27_	*Vibrio natriegens*	OP703612.1
*Vibrio* ChromoSelect agar	*S. aurata* intestines	Turquoise	SA_5_	*Photobacterium damselae*	OP704023.1
	*S. aurata* intestines	Light green with a green center	SA_7_	*Staphylococcus epidermidis*	OP704017.1
	*S. aurata* intestines	Colorless	SA_9_	*Bacillus cereus*	OP704016.1
	*S. aurata* gills	Light green with a green center	SA_11_	*Vibrio natriegens*	OP704027.1
	*S. aurata* gills	Colorless	SA_13_	*Vibrio natriegens*	OP704012.1
	*S. aurata* gills	Pink	SA_15_	*Aeromonas veronii*	OP703806.1
	*S. aurata* intestines	Green	SA_25_	*Aeromonas veronii*	OP704013.1
	*S. aurata* muscles	Blue	SA_26_	*Vibrio harveyi*	OP703738.1
	*S. aurata* muscles	Colorless	SA_31_	*Aeromonas veronii*	OP704014.1

**Table 2:** Exoenzyme production by the identified bacterial strains.

Code	Strains	DNase	Lipase	Lecithinase	Caseinase	Hemolysis	Amylase	Gelatinase
P_1_	*V.* *natriegens*	+	+	+	+	−	+	−
P_14_	*V.* *natriegens*	+	+	−	−	−	+	−
SA_11_	*V.* *natriegens*	+	+	−	−	−	+	−
SA_13_	*V.* *natriegens*	−	+	+	+	−	+	+
SA_27_	*V.* *natriegens*	+	+	−	−	−	+	−
P_9_	*V.* harveyi *	+	+	+	+	+	+	+
SA_26_	*V.* *harveyi*	−	+	−	+	−	+	+
P_2_	*V.* alginolyticus *	+	+	+	+	+	+	+
P_12_	*V.* *hyugaensis*	+	+	−	−	−	+	−
SA_1_	*A.* *veronii*	−	−	−	+	−	+	+
SA_15_	*A.* veronii *	+	+	+	+	+	+	+
SA_17_	*A.* veronii *	+	+	+	+	+	+	−
SA_25_	*A.* veronii *	+	−	+	+	+	+	+
SA_31_	*A.* veronii *	+	−	+	+	+	+	−
SA_3_	*P.* piscicida *	−	+	−	−	+	+	−
SA_5_	*P.* damselae *	−	+	−	−	+	+	−
P_5_	*M.* *morganii*	+	−	−	−	−	+	−
P_13_	*S.* indica *	+	+	+	+	+	+	+
SA_9_	*B.* cereus *	+	+	+	+	+	+	−
SA_7_	*S.* *epidermidis*	−	+	+	+	−	+	+
SA_21_	*V.* fluvialis *	+	+	+	+	+	+	+

(+): positive test, (−): negative test; * β-hemolytic strain.

**Table 3:** Distribution of ARI in the different bacterial populations identified in this study.

Microorganisms Tested	Antibiotic Resistance Index (ARI)
*Vibrio* spp. (n = 9)	0.542
*Aeromonas* spp. (n = 5)	0.553
All Gram-negative	0.544
All Gram-positive	0.462

**Table 4:** Exopolysaccharides production (slime) on Congo red agar, and ability to adhere to a glass surface (Wolfe test).

Code	Bacteria Tested	Slime Production on CRA		Wolfe Test
		Morphotype	Interpretation	
P_1_	*V. natriegens*	Red	Non producer	++
P_14_	*V. natriegens*	Black	Producer	+
SA_11_	*V. natriegens*	Black	Producer	++
SA_13_	*V. natriegens*	Orange	Non producer	++
SA_27_	*V. natriegens*	Black	Producer	++
P_9_	*V. harveyi*	Black	Producer	++
SA_26_	*V. harveyi*	Red	Non producer	+
P_2_	*V. alginolyticus*	Bordeaux	Non producer	++
P_12_	*V. hyugaensis*	Bordeaux	Non producer	+
SA_1_	*A. veronii*	Black	Producer	+
SA_15_	*A. veronii*	Bordeaux	Non producer	++
SA_17_	*A. veronii*	White with red center	Non producer	+
SA_25_	*A. veronii*	Black	Producer	++
SA_31_	*A. veronii*	Black gray	Non producer	+
SA_3_	*P. piscicida*	Red	Non producer	+
SA_5_	*P. damselae*	Red	Non producer	−
P_5_	*M. morganii*	Red	Non producer	+
P_13_	*S. indica*	Bordeaux	Non producer	++
SA_9_	*B. cereus*	Bordeaux	Non producer	++
SA_7_	*S. epidermidis*	Red	Non producer	+
SA_21_	*V. fluvialis*	Bordeaux	Non producer	+

(++): large pellicule formation; (+) weak pellicule formation; (−) no pellicule formation.

**Table 5:** Biofilm formation by the 21 identified bacterial strains from *S. aurata* and *P. indicus* on polystyrene 96-well plates, as well as glass and plastic abiotic surfaces.

Code	Bacteria Tested	Polystyrene *		Glass **		Plastic **	
		OD_595nm_ ± SD	Interpretation	OD_595nm_ ± SD	Interpretation	OD_595nm_ ± SD	Interpretation
P_1_	*V. natriegens*	0.360 ± 0.024	(−); Non biofilm forming	0.102 ± 0.002	(+); Weakly adherent	0.152 ± 0.036	(++); Moderately adherent
P_14_	*V. natriegens*	0.746 ± 0.002	(−); Non biofilm forming	0.114 ± 0.006	(+); Weakly adherent	0.412 ± 0.022	(+++), Strongly adherent
SA_11_	*V. natriegens*	2.029 ± 0.166	(++); Medium biofilm forming	0.070 ± 0.004	(−); Non adherent	0.081 ± 0.013	(+); Weakly adherent
SA_13_	*V. natriegens*	0.976 ± 0.061	(−); Non biofilm forming	0.108 ± 0.009	(+); Weakly adherent	0.387 ± 0.514	(+++), Strongly adherent
SA_27_	*V. natriegens*	0.599 ± 0.026	(−); Non biofilm forming	0.189 ± 0.010	(++); Moderately adherent	0.148 ± 0.008	(++); Moderately adherent
P_9_	*V. harveyi*	1.492 ± 0.119	(+); Weak biofilm forming	0.101 ± 0.027	(+); Weakly adherent	0.081 ± 0.017	(+); Weakly adherent
SA_26_	*V. harveyi*	0.981 ± 0.178	(−); Non biofilm forming	0.245 ± 0.023	(++); Moderately adherent	0.219 ± 0.061	(++); Moderately adherent
P_2_	*V. alginolyticus*	2.070 ± 0.076	(++); Medium biofilm forming	0.188 ± 0.045	(++); Moderately adherent	0.116 ± 0.033	(+); Weakly adherent
P_12_	*V. hyuganesis*	2.029 ± 0.206	(++); Medium biofilm forming	0.075 ± 0.008	(−); Non adherent	0.067 ± 0.009	(−); Non adherent
SA_1_	*A. veronii*	1.505 ± 0.072	(+); Weak biofilm forming	0.112 ± 0.007	(+); Weakly adherent	0.099 ± 0.022	(+); Weakly adherent
SA_15_	*A. veronii*	1.500 ± 0.118	(+); Weak biofilm forming	0.140 ± 0.013	(+); Weakly adherent	0.055 ± 0.005	(−); Non adherent
SA_17_	*A. veronii*	0.835 ± 0.055	(−); Non biofilm forming	0.091 ± 0.016	(+); Weakly adherent	0.077 ± 0.005	(+); Weakly adherent
SA_25_	*A. veronii*	0.351 ± 0.021	(−); Non biofilm forming	0.158 ± 0.021	(+); Weakly adherent	0.060 ± 0.004	(−); Non adherent
SA_31_	*A. veronii*	1.214 ± 0.216	(+); Weak biofilm forming	0.142 ± 0.010	(+); Weakly adherent	0.099 ± 0.005	(+); Weakly adherent
SA_3_	*P. piscicida*	2.792 ± 0.244	(++); Medium biofilm forming	0.246 ± 0.027	(++); Moderately adherent	0.077 ± 0.001	(+); Weakly adherent
SA_5_	*P. damselae*	0.410 ± 0.028	(−); Non biofilm forming	0.109 ± 0.010	(+); Weakly adherent	0.118 ± 0.019	(+); Weakly adherent
P_13_	*S. indica*	0.505 ± 0.078	(−); Non biofilm forming	0.110 ± 0.007	(+); Weakly adherent	0.418 ± 0.004	(+++), Strongly adherent
P_5_	*M. morganii*	0.782 ± 0.053	(−); Non biofilm forming	0.114 ± 0.012	(+); Weakly adherent	0.133 ± 0.009	(+); Weakly adherent
SA_9_	*B. cereus*	2.525 ± 0.210	(++); Medium biofilm forming	0.313 ± 0.061	(++); Moderately adherent	0.151 ± 0.025	(++); Moderately adherent
SA_7_	*S. epidermidis*	0.781 ± 0.023	(−); Non biofilm forming	0.114 ± 0.009	(+); Weakly adherent	0.182 ± 0.010	(++); Moderately adherent
SA_21_	*V. fluvialis*	0.307 ± 0.011	(−); Non biofilm forming	0.116 ± 0.012	(+); Weakly adherent	0.078 ± 0.018	(+); Weakly adherent

* Interpretation of biofilm formed on polystyrene surface [33]: (−) non biofilm forming OD595 ≤ 1; (+) weak biofilm forming 1 < OD595 ≤ 2; (++) medium biofilm forming 2 < OD595 ≤ 3; (+++) strong biofilm forming OD595 > 3. ** Interpretation of biofilm formed on glass and plastic surfaces [37]: nonadherent (0) OD ≤ ODc; weakly adherent (+) ODc < OD ≤ 2 × ODc; moderately adherent (++) 2 × ODc < OD ≤ 4 × ODc; strongly adherent (+++) 4 × ODc < OD.

## Data Availability

All data are presented in the manuscript.

## Supplementary Materials

The following supporting information can be downloaded at https://www.mdpi.com/article/10.3390/life13020548/s1. Table S1. Antibiotic susceptibility of 18 Gram negative bacteria to 26 antibiotics and MARI calculation; Table S2. Antibiotic susceptibility of *V. fluvialis*, *B. cereus* and *S. epidermidis* to 18 antibiotics and MARI calculation.
