# Supplementary material for: Molecular Identification of Bacteria Isolated from Marketed Sparus aurata and Penaeus indicus Sea Products: Antibiotic Resistance Profiling and Evaluation of Biofilm Formation
Source: Life (Basel). 2023 Feb 16;13(2):548. doi: 10.3390/life13020548 (PMC9963372; doi:10.3390/life13020548)
Supplement: Supplementary file 1 [file life-13-00548-s001.zip › life-2169853-supplementary.pdf]

life

Molecular Identification of Bacteria Isolated from Marketed *Sparus aurata* and *Penaeus indicus* Sea Products: Antibiotic Resistance Profiling and Evaluation of Biofilm Formation

Mohammad A. Abdulhakeem [1], Mousa Alreshidi [1,2,*], Fevzi Bardakci [1], Walid Sabri Hamadou [1], Vincenzo De Feo [3], Emira Noumi [1,4,*] and Mejdi Snoussi [1,4]

[1] Department of Biology, College of Science, University of Ha'il, Ha'il P.O. Box 2440, Saudi Arabia
[2] Molecular Diagnostics and Personalized Therapeutics Unit, University of Hail, Hail P.O. Box 2440, Saudi Arabia
[3] Department of Pharmacy, University of Salerno, Via Giovanni Paolo II, 132, Fisciano, 84084 Salerno, Italy
[4] Laboratory of Genetics, Biodiversity and Valorization of Bio-Resources (LR11ES41), Higher Institute of Biotechnology of Monastir, University of Monastir, Avenue Tahar Haddad, BP74, Monastir 5000, Tunisia
* Correspondence: mo.alreshidi@uoh.edu.sa (M.A.); eb.noumi@uoh.edu.sa (E.N.)

Supplementary material Table S1. Antibiotic susceptibility of 18 Gram negative bacteria to 26 antibiotics and MARI calculation.

Code	Bacteria Tested	1	2	3	4	5	6	7	8	9	10	11	12	13	14	15	16	17	18	19	20	21	22	23	24	25	26	MARI
P_{1}	*V. natrigens*	S	S	R	R	S	S	R	R	R	S	R	R	R	R	R	R	R	R	S	R	S	S	S	R	R	S	0.615
P_{14}	*V. natrigens*	S	S	R	R	R	R	R	R	R	I	R	R	R	R	R	R	R	I	S	S	S	S	R	R	R	S	0.423
SA_{11}	*V. natrigens*	S	S	R	S	R	S	R	R	R	I	R	R	R	R	R	R	R	R	S	R	S	S	R	R	R	S	0.538
SA_{13}	*V. natrigens*	S	S	S	R	I	I	S	S	S	R	S	R	R	R	I	I	R	R	I	R	S	I	R	R	R	S	0.615
SA_{27}	*V. natrigens*	S	S	R	I	R	I	R	R	R	I	R	S	R	R	R	I	R	R	S	R	R	S	S	R	S	S	0.653
P_{9}	*V. harveyi*	S	S	R	S	I	S	S	R	R	S	R	S	R	R	S	I	R	R	I	R	S	S	S	R	S	S	0.5
SA_{26}	*V. harveyi*	R	S	R	S	I	R	I	R	I	S	R	R	R	R	R	I	R	I	S	R	S	S	R	R	S	S	0.384
P_{2}	*V. alginolyticus*	S	S	R	I	R	R	R	R	R	R	R	R	R	R	I	R	R	R	S	R	S	S	S	R	R	S	0.615
P_{12}	*V. hyuganesis*	S	S	R	S	R	S	R	R	R	S	R	S	R	R	I	S	R	S	S	R	S	S	S	R	S	S	0.423
**Total R (%)**		11	0	89	33	56	33	67	89	78	22	89	67	100	100	56	44	100	67	0	89	11	0	44	100	56	0	
**Total I (%)**		0	0	0	22	33	22	22	0	11	33	0	0	0	0	33	44	0	22	33	0	0	11	0	0	0	0	
**Total S (%)**		89	100	11	44	11	44	11	11	11	44	11	33	0	0	11	11	0	11	77	11	89	89	56	0	44	100	
SA_{1}	*A. veronii*	I	S	R	I	R	R	R	R	I	S	R	R	R	R	R	R	R	R	I	R	S	S	R	R	R	S	0.653
SA_{15}	*A. veronii*	R	S	S	S	R	I	S	R	R	R	R	R	R	R	I	S	R	R	S	R	S	S	S	R	S	R	0.538
SA_{17}	*A. veronii*	R	S	S	S	I	S	S	R	R	I	R	R	R	R	R	I	R	S	R	R	S	S	S	R	S	S	0.461
SA_{25}	*A. veronii*	S	S	R	R	R	R	I	R	R	R	R	R	R	R	R	R	R	R	S	R	S	S	S	R	R	S	0.692
SA_{31}	*A. veronii*	S	S	S	S	R	I	I	R	R	R	R	R	R	R	R	R	R	R	S	R	S	I	S	R	S	I	0.538
**Total R (%)**		40	0	40	20	80	40	20	100	80	60	100	100	100	100	80	60	100	80	20	100	0	0	20	100	40	20	
**Total I (%)**		20	0	60	20	20	40	40	0	20	20	0	0	0	0	20	20	0	0	20	0	0	20	0	0	0	20	
**Total S (%)**		40	100	0	60	0	20	40	0	0	20	0	0	0	0	0	20	0	20	60	0	100	80	100	0	60	60	
SA_{3}	*P. piscicida*	S	S	R	S	S	I	S	R	R	S	R	R	R	R	R	R	R	R	I	R	S	S	I	R	R	I	0.538
SA_{5}	*P. damselae*	S	S	R	I	I	R	R	R	R	S	R	R	R	R	R	R	R	S	I	R	S	S	R	R	R	S	0.615
P_{13}	*S. indica*	I	I	R	R	R	R	R	R	R	I	R	R	R	R	S	R	R	R	S	R	S	S	S	R	R	S	0.653
P_{5}	*M. morganii*	S	S	R	R	I	R	R	R	R	S	R	R	R	R	S	I	R	I	I	S	S	S	S	R	S	S	0.423
**Total R (%)**		0	0	100	50	25	75	75	100	100	0	100	100	100	100	50	75	100	50	0	75	0	0	25	100	75	0	
**Total I (%)**		25	25	0	25	50	25	0	0	0	25	0	0	0	0	0	25	0	25	75	0	0	0	25	0	0	25	
**Total S (%)**		75	75	0	25	25	0	25	0	0	75	0	0	0	0	50	0	0	25	25	25	100	100	50	0	25	75	
**R for All G-**		17	0	78	33	56	44	56	94	83	28	94	83	100	100	61	56	100	67	6	89	6	0	33	100	61	6	

1-NA 2-NOR 3-TMO 4-FOS 5-ETP 6-CIP 7-F 8-AK 9-GEN 10-TET 11-AMP 12-MXF 13-MEM 14-TIC 15-PPT 16-CTX 17-TGC 18-E 19-C 20-AUG 21-TOB 22-SXT 23-CZD 24-CPN 25-CST 26-NET

Supplementary material Table S2. Antibiotic susceptibility of *V. fluvialis, B. cereus* and *S. epidermidis* to 18 antibiotics and MARI calculation.

Code	Bacteria	1	2	3	4	5	6	7	8	9	10	11	12	13	14	15	16	17	18	MARI
SA_7	*S. epidermidis*	S	S	I	I	I	I	R	R	I	R	S	R	S	S	R	I	R	S	0.333
SA_{21}	*V. fluvialis*	S	S	R	S	I	R	R	R	S	S	S	S	I	R	S	I	S	S	0.277
SA9	*B. cereus*	S	S	I	S	R	S	R	R	I	R	S	R	S	R	R	R	R	S	0.5
Total R (%)		0	0	33	0	33	33	100	100	0	67	0	67	0	67	67	33	67	0	
Total I (%)		0	0	67	33	67	33	0	0	67	0	0	0	33	0	0	67	0	0	
Total S (%)		100	100	0	67	0	33	0	0	33	33	100	33	67	33	33	0	33	100	
1-NOR 2-FOS 3-F 4-AK 5-GEN 6-TET7-AMP 8-MXF 9-RAM 10-PTN 11-E 12-AUG 13-TOB 14-SXT 15-LZD 16-CPN																				
